# Clinical outcome of supervised pulmonary telerehabilitation program among adult patients with post‐acute COVID‐19 symptoms (PACS): A case series

**DOI:** 10.1002/rcr2.1187

**Published:** 2023-07-05

**Authors:** Nikko John Dalisay, Bernice Ong ‐ dela Cruz, Percival Punzal, Ma. Encarnita Limpin

**Affiliations:** ^1^ Division of Pulmonary and Critical Care Medicine Philippine Heart Center Quezon City Philippines

**Keywords:** post‐acute COVID‐19 symptoms, pulmonary rehabilitation, telerehabilitation

## Abstract

Patients with post‐acute COVID‐19 symptoms (PACS) can present with significant sequela due to the complex systemic effects of COVID‐19 infection. Most affected patients have persistent symptoms for 3–12 months after recovery from the acute phase of COVID‐19. Dyspnea affecting activities of daily living is one of the most challenging symptoms and has led to an influx of pulmonary rehabilitation (PR) demand. Here we report the outcome of nine subjects with PACS who underwent 24 sessions of supervised pulmonary telerehabilitation. An improvised telerehabilitation PR was formulated to accommodate home confinement during the pandemic. Exercise capacity and pulmonary function were assessed using a cardiopulmonary exercise test, pulmonary function test, and St. George Respiratory Questionnaire (SGRQ). The clinical outcome shows improved exercise capacity on the 6‐minute walk test for all patients, and most had improvement in VO_2_ peak and SGRQ. Seven patients improved in forced vital capacity and six in forced expiratory volume. PR is a comprehensive intervention for patients with chronic obstructive disease aimed at alleviating pulmonary symptoms and improving functional capacity. In this case series, we report its usefulness in patients with PACS and its feasibility when delivered as a supervised telerehabilitation program. Further support for the effectiveness of PR patients with PACS is mandated.

## Graphical abstract

Here we report the outcome of nine subjects with post‐acute COVID‐19 symptoms (PACS) who underwent 24 sessions of supervised pulmonary telerehabilitation. An improvised telerehabilitation PR was formulated to accommodate home confinement during the pandemic. Exercise capacity and pulmonary function were assessed using a cardiopulmonary exercise test, pulmonary function test, and St. George Respiratory Questionnaire (SGRQ). The clinical outcome shows improved exercise capacity on the 6‐minute walk test for all patients, and most had improvement in VO_2_ peak and SGRQ. Seven patients improved in forced vital capacity and six in forced expiratory volume. This case series shows the clinical impact and benefit of pulmonary telerehabilitation in a resource‐limited setting.

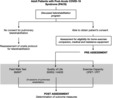

## INTRODUCTION

The coronavirus disease (COVID‐19) is an infectious disease caused by a new strain of severe acute respiratory syndrome coronavirus (SARS‐CoV‐2).^1^ Approximately 10%–15% of cases progress to severe illness, and about 5% become critically ill. Patients with COVID‐19 pneumonia typically recover from the disease after 2–6 weeks. The World Health Organization recognizes that respiratory symptoms such as cough and shortness of breath may recur for weeks or months following initial recovery in some patients. This phenomenon can affect those with seemingly mild diseases and those without co‐morbidities.^2^ The presence of lingering symptoms does not render them infectious. However, the lingering symptoms may impact their health, quality of life, and ability to perform tasks.

The extent of the impairment and disability brought by COVID‐19 is unknown. Rehabilitation is ‘a set of interventions designed to reduce disability and optimize functioning in individuals with health conditions in interaction with their environment’.^3^ A supervised pulmonary telerehabilitation program is a subset of telemedicine primarily using electronic means by remotely assessing, evaluating, and monitoring therapy to provide rehabilitation care to various locations.^4^ The feasibility of telerehabilitation in a developing Asian country has led to the adoption of a paradigm protocol during the previous SARS pandemic composed of pulmonary rehabilitation for 1.0–1.5 h duration four times a week for 6 weeks on a submaximal training with inclusion of upper and lower extremity exercises and relevant health education.^5^

Responsive to growing needs due to healthcare workers affected by COVID‐19, the section of Pulmonary Rehabilitation at the Philippine Heart Center started conducting telerehabilitation for healthcare workers needing therapy and patients from remote areas of the country.

## CASE SERIES

### Telerehabilitation intervention

**FIGURE 1:**
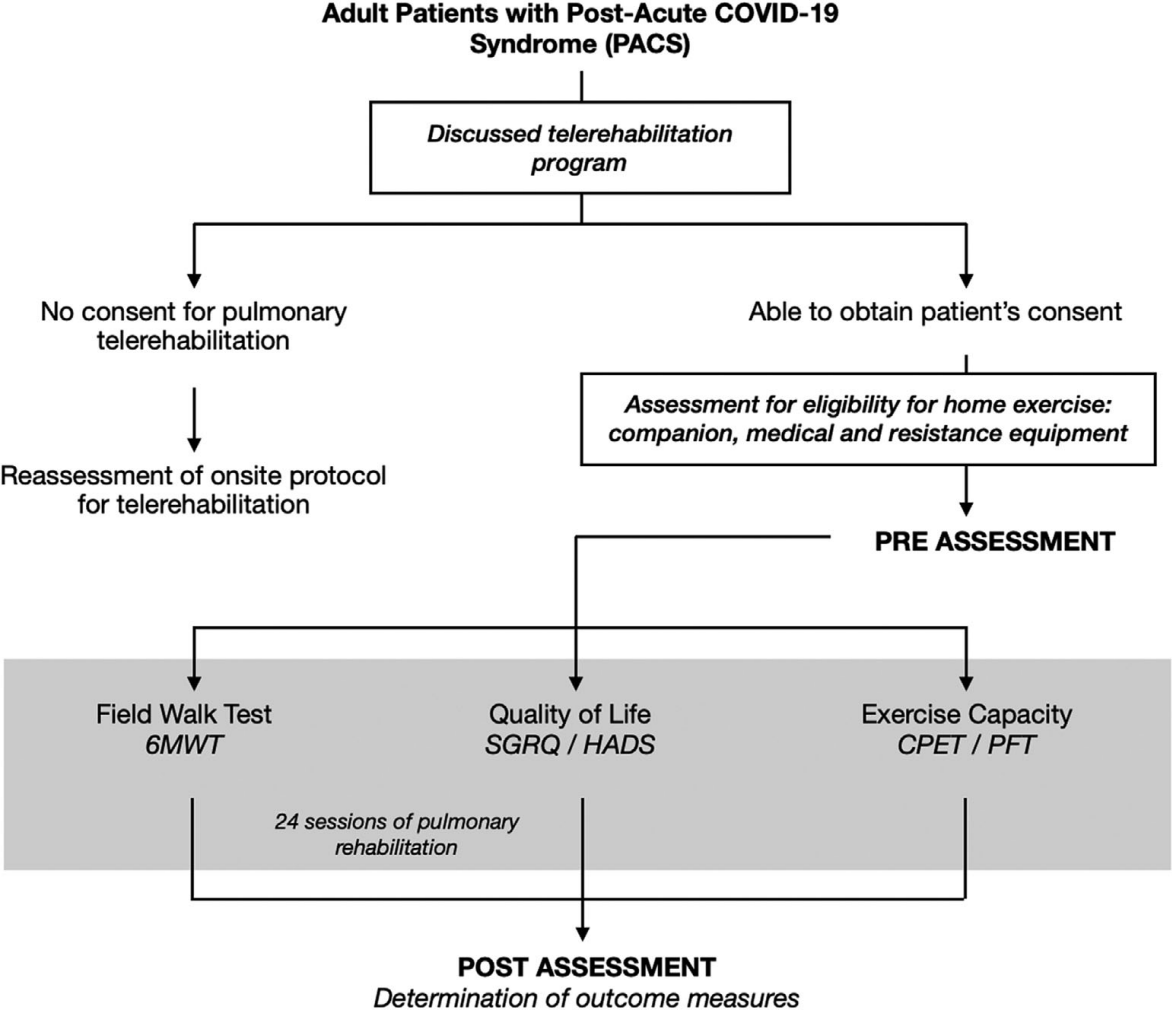
Pathway of pulmonary rehabilitation program during the pandemic.

The Philippine Heart Center opened the supervised pulmonary telerehabilitation program to cater to patients previously infected by COVID‐19 (Figure 1). A total of 37 patients were identified in the pulmonary rehabilitation program from March 2020 to 2022.

To be able to join the telerehabilitation program, patients were required to meet the following pre‐requisite for the program: (1) provide informed consent, (2) have a primary care giver who will supervise during the telerehabilitation, (3) have direct access to a local health facility, (4) have essential medical equipment such as pulse oximeter and blood pressure monitor, (5) have dumbbells or improvised resistance equipment such as water bottles and lastly, and (6) have an internet connection. The leading platform to be utilized was Zoom meetings. Sessions lasted approximately 1–1 ½ h, at 2–3 sessions per week. The sessions were recorded for documentation and legal purposes. The data was to be discarded 2 months after the participant's completion.

The main reasons patients did not meet the pre‐requisite to be included in the program were lack of consent, a lack of primary caregivers that will set up and supervise the telerehabilitation sessions or lack of funding to provide the equipment required.

Thus, only 13 patients met the inclusion criteria for the hybrid telerehabilitation program and were enrolled in the program.

Nine^6^ patients undertook the complete telerehabilitation sessions and are presented in this case series. Reasons for drop‐out were mainly conflict of schedule for completing the program during office hours due to work commitment.

The clinical research fellow supervising the telerehabilitation program completed an initial assessment of the patients upon enrollment in the program. It explained the program's concept, procedures, advantages, and modifications from the usual pulmonary rehabilitation, and consent was obtained upon discussion of patient safety. Each patient underwent an on‐site pre‐assessment to measure the minimum clinically significant difference (MCID) for outcome measures. It included quality of life measurement with the St. George's Respiratory Questionnaire (SGRQ) and Hospital Anxiety and Depression Scale (HADS), measurements of physical exertion such as the Six‐Minute Walk Test (6MWT) and VO_2_ Max from cardiopulmonary exercise test (CPET). An on‐site post‐assessment of respiratory symptoms and exercise capacity was done upon completion of the 24 sessions of exercise therapy. In between sessions, health education in nutrition, breathing exercises, management of acute exacerbation and health management among primary caregivers was an essential part of PR. Topics included breathing exercises, handling exacerbations, and nutrition.

During the exercise sessions, a companion at home monitored the patient's blood pressure, heart rate, and oxygen saturation. At the end of each session, a Modified Borg Scale was used to document the severity of perceived muscle fatigue and breathlessness, which was utilized in necessary adjustments regarding workload and the number of repetitions.

### Patient information and clinical findings

Table 1 summarizes the demographic and clinical profiles of the nine patients. The patients enrolled for the supervised pulmonary telerehabilitation program of the Philippine Heart Center were previous COVID‐19 patients with a COVID‐19 illness course ranging from mild to critical cases, however, most patients^3^ had had a moderate COVID‐19 illness. Most of the patients were females.^5^ The ages varied widely from young adults to elderly patients (33–78 years). Age was a factor in the duration of the completion of PR, and younger adults tended to take longer to complete the program. Most patients were obese with pre‐existing co‐morbidities such as hypertension, diabetes mellitus, and bronchial asthma.

**TABLE 1:** Demographic and clinical profiles of the nine patients.

	Case 1	Case 2	Case 3	Case 4	Case 5	Case 6	Case 7	Case 8	Case 9
Age	46	77	70	78	33	36	50	38	38
Sex	F	M	M	F	M	F	F	F	F
BMI	37.9	20.2	22.7	24.6	38	39.6	38.4	34	31.6
Co‐Morbidities	DM, HTN	COPD	COPD, CAD	HTN	BA	HTN. BA, OSA	DM, HTN, BA	BA	HTN
COVID‐19	Severe	Severe	Moderate	Critical	Moderate	Moderate	Moderate	Mild	Moderate
Severity Duration of PR	65 days	76 days	180 days	119 days	239 days	256 days	150 days	81 days	238 days

### Telerehabilitation outcomes

Parameters were assessed post‐rehabilitation. In terms of exercise capacity, most of the patients^7^ showed a significant improvement in the VO_2_ peak, and all^6^ of the patients had a substantial change in terms of walk distance post‐assessment concerning the minimum clinically important difference of more than 30 m.^8^ In terms of quality of life, more than half had a substantial change in MCID. In addition, anxiety and depression show a decreasing trend post‐pulmonary rehabilitation. Lung function also showed remarkable results; most^9^ had increasing FVC, and six had improvement on FEV1, respectively, as summarized in Table 2.

**TABLE 2:** Summary of changes in exercise capacity and pulmonary function prior and after the pulmonary telerehabilitation.

Variables	Case 1	Case 2	Case 3	Case 4	Case 5	Case 6	Case 7	Case 8	Case 9
VO_2_ peak (mL/kg/min)									
Baseline	11.3	14.6	7.7	11	22.9	13.3	15.9	17.5	20.6
After PR	11.6	17	9.3	12.7	20.2	14.6	18.1	23.6	17.5
*Change rate (%)*	+2.6	+16.4	+20.8	+15.5	‐11.8	+9.8	+13.8	+34.9	−15.0
VO_2_ peak based on % predicted value									
Baseline	59	48	30	61	76	63	81	77	77
After PR	63	64	35	70	60	70	93	95	70
*Difference (%)*	+4	+16	+5	+9	‐16	+7	+12	+18	−3
6 MWT (m)									
Baseline	300	200	242	122	392	274	304	479	345
After PR	360	360	400	180	458	415	408	518	431
*Change rate (%)*	+20	+80	+65.2	+47.5	+16.8	+51.5	+34.2	+8.14	+24.9
HADS									
*Anxiety*									
Baseline	6	13	14	2	9	12	9	11	7
After PR	4	5	8	1	2	1	5	6	3
*Depression*									
Baseline	5	12	13	2	5	9	3	9	6
After PR	2	2	8	0	3	1	6	4	3
SGRQ									
Baseline	23.6	14.12	55.37	38.45	32.02	59.07	51.17	48.4	12.53
After PR	23.77	12.14	9.02	16.2	29.92	30.82	18.89	28.31	4.17
*Difference*	−0.17	+2.02	+46.35	+22.25	+2.08	+28.25	+32.28	+23.09	+8.36
FVC (%)									
Baseline	85	60	57	75	100	94	51	90	83
After PR	87	88	63	76	97	86	73	99	87
*Change rate (%)*	+2.4	+46.7	+10.5	+1.3	−3	‐8.5	+43.1	+10	+4.8
FEV_1_ (%)									
Baseline	90	48	32	88	102	94	57	86	91
After PR	92	59	35	94	98	88	73	89	89
*Change rate (%)*	+2.2	+22.9	+9.4	+6.8	−3.9	−6.4	+28.1	+3.5	−2.2

Moreover, there were no reported injuries (falls, trauma or fracture) or further complications (shortness of breath, chest pain or loss of consciousness) at any part of the program. Technical difficulties were quickly addressed since most patients were being assisted remotely at home.

## DISCUSSION

The COVID‐19 pandemic has led to a surge in demand for pulmonary rehabilitation, specifically in low‐income countries, and with the shift from traditional rehabilitation to social distancing. Integrating a telerehabilitation program into a facility‐based rehabilitation unit has resolved this problem.^9^ Several factors that may hinder the implementation of such program are the availability of resources such as an internet connection, and the Philippines' probable limitation is its meagre connectivity. The results presented here show favourable outcomes, similar to patients with COPD on pulmonary telerehabilitation regarding dyspnea and exercise capacity.^7^

To our knowledge, this is the first study to document cardiopulmonary fitness using VO_2_ peak among patients who had undergone pulmonary telerehabilitation. The result shows a mean increase of 12.9% in VO_2_ peak with a clinical benefit of all‐cause and disease‐specific mortality.^6^ Regarding 6 MWT, the field walk test documented a mean of 38.69% change. The increased 6 MWT distance post‐rehabilitation has been established objectively, as evidenced by the significant outcome.

The outcome of anxiety and depression are similar to an onsite pulmonary rehabilitation with a proposed MCID for HADS‐Anxiety of 1.7 and HADS‐Depression of 1.5, which were adequately met in this report and are often equivalent to higher levels of self‐efficacy.^10^ Regarding FEV1 and FVC, most patients had improved pulmonary function post‐telerehabilitation. Moreover, due to conflicting study results, robust evidence must be established on the clinical implication of pulmonary rehabilitation on improving lung volume.^11^ In addition, the effect of body mass index (BMI) on clinical improvement is positively associated with the obesity paradox; an increase in BMI may have a protective effect against mortality. However, several confounders, such as disease severity, smoking history, and body composition, may significantly affect the protective role of obesity.^12^

In this case series, pulmonary telerehabilitation improved pulmonary function, exercise capacity, and quality of life for the patients. Regardless of the quarantine limitations preventing onsite pulmonary rehabilitation, this protocol of pulmonary telerehabilitation can be a promising innovation even in the post‐pandemic period. Apart from the clinical implication, this telemedicine branch's economic impact has proven its effectiveness in decreasing costs, improving efficiency, and increasing healthcare delivery access.^13^

In conclusion, smartphones have become available and easy to use among Filipinos, and pulmonary telerehabilitation could be a viable alternative in remote areas of the Philippine archipelago.^14^ Exercise therapy is the cornerstone of pulmonary rehabilitation. In addition, telerehabilitation can also adapt to the other components of pulmonary rehabilitation, such as patient education, nutritional support, and psychosocial support.^15^ This case series shows the clinical impact of pulmonary telerehabilitation in a resource‐limited setting.

## AUTHOR CONTRIBUTIONS

The authors confirm their contribution to the paper: study conception and design: Nikko John Dalisay, Bernice Ong ‐ dela Cruz: Data collection: Nikko John Dalisay; analysis and interpretation of results: Nikko John Dalisay, Percival Punzal: Draft manuscript preparation: Nikko John Dalisay, Ma. Encarnita Limpin. All authors reviewed the results and approved the final version of the manuscript.

## CONFLICT OF INTEREST STATEMENT

None declared.

## ETHICS STATEMENT

The study complies with the ethical principles outlined in the Declaration of Helsinki and the National Ethical Guidelines for Health and Health‐Related Research of 2017. During the study initiation, the Philippine Heart Center Institutional Ethics Review Board (PHC IERB at 028925 2401 local 3899) reviewed and approved the study protocol, informed consent, and subsequent amendments.

## Data Availability

Data openly available in a public repository that issues datasets with DOIs.
